# Editorial: Causes of Oocyte Aneuploidy and Infertility in Advanced Maternal Age and Emerging Therapeutic Approaches

**DOI:** 10.3389/fendo.2021.652990

**Published:** 2021-02-23

**Authors:** Lori R. Bernstein, Nathan R. Treff

**Affiliations:** ^1^ Pregmama, LLC, Gaithersburg, MD, United States; ^2^ Department of Epidemiology and Public Health, University of Maryland School of Medicine, Baltimore, MD, United States; ^3^ Department of Veterinary Integrative Biosciences, Texas A&M College of Veterinary Medicine, College Station, TX, United States; ^4^ Genomic Prediction Inc., North Brunswick, NJ, United States; ^5^ Genomic Prediction Clinical Laboratory, North Brunswick, NJ, United States

**Keywords:** infertility, advanced maternal age, aneuploidy, preimplantation genetic testing, reactive oxygen species, endometriosis

Women of advanced maternal age (AMA, age ≥ 35) experience elevated rates of infertility, miscarriages and trisomic pregnancies (Ubaldi et al.). They also exhibit diminished ovarian reserve (DOR), as well as poor ovarian responses (POR) to gonadotropin stimulation in assisted reproduction (Fuentes et al.). Oocyte and embryo aneuploidy are primary causes of declining oocyte and embryo quality and diminished rates of pregnancy and livebirth in AMA women. Mechanisms that underlie these processes are thought to include cohesin dysfunction, telomere shortening, spindle instability, aberrant checkpoint control, reductions in rates of preimplantation embryo development to the blastocyst stage, age-related hormonal aberrations, and mitochondrial dysfunction (Ubaldi et al.; Fuentes et al.) (1). Some factors that reduce oocyte quality and embryo quality in AMA women may also be at play in the pathogenesis of endometriosis (Máté et al.). Current and emerging technological advances continue to be developed to treat and even prevent oocyte aneuploidy in patients with diminished oocyte and embryo quality. Here present an editorial summary of these themes in this article collection, along with some recent data drawn from additional relevant papers.

## Causative Factors

There have been recent advances in understanding molecular determinants of declining ovarian reserve and oocyte and embryo quality. Patterns of oocyte epigenetic modifications in oocytes, including DNA methylation and histone modifications in chromatin, undergo distinctive changes with maternal age (Chamani and Keefe). These changes may have causative roles in the increased aneuploidy of oocytes in AMA women. There also is mounting evidence that reactive oxygen species (ROS) adversely impact oocyte and embryo quality both in AMA women, and in women with endometriosis (Máté et al.) (2). ROS from endogenous and exogenous sources over time may be drivers of molecular damage to oocyte and embryo chromosome segregation apparati, as well as the molecular components of other organelles (e.g., mitochondria) in aging women, causing aneuploidy and other manifestations of poor oocyte quality (2). Retrograde menstruation occurs in women with endometriosis. This brings menstrual blood into proximity to the fallopian tubes and ovaries. As the refluxed blood undergoes natural degradation, intracellular contents are released from red blood cells. This markedly increases local concentrations of iron that drive formation of the powerful ROS hydroxyl radical, potentially damaging cellular structures of oocytes and embryos residing in the ovaries and/or fallopian tubes to worsen oocyte and embryo quality (Máté et al.).

Hormonal changes that occur with age are postulated to have significant detrimental effects on fertility in AMA women. Androgens induce survival and growth of small follicles and induce expression of FSH receptors in granulosa cells (3, 4). AMA and DOR/POR are associated with significant declines in serum androgens and sex hormone binding globulin (SHBG) among several POSOIDON study groups of infertile women with POR (Fuentes et al.). While correlative, these data are consistent with the notion that declining androgen production that occurs with age and follicular depletion may contribute to worsening fertility in POR patients.

FSH elevation has been shown to reduce endometrial receptivity, lowering implantation and pregnancy rates (5, 6). In studies employing the SAMP8 mouse model of female reproductive aging, midlife female mice have elevated FSH, higher rates of oocyte chromosome and spindle misalignments, diminished yield of ovulated eggs, and lower fertility, than young female mice (7). Treating the midlife mice with equine chorionic gonadotropin (eCG), a hormone with high FSH activity, lowers yield of viable ovulated oocytes, and increases rates of oocyte chromosome and spindle misalignments.

Activin is an endogenous molecule that induces elevation of serum FSH (8). The soluble recombinant fusion protein ActRIIB:Fc is termed an activin decoy receptor because it binds and sequesters activin, preventing its binding to cell surface activin receptors (9). Administration of ActRIIB:Fc to midlife SAMP8 female mice lowers their serum FSH to young levels, decreases rates of oocyte chromosome and spindle misalignments, raises yields of viable ovulated oocytes, and increases their fertility (1). These data support the hypothesis that FSH elevation and/or other activities of the activin signaling pathway promote oocyte aneuploidy and infertility in AMA mice.

## Current and Emerging Methods for Improving AMA Reproductive Success Stimulation Protocols

Various assisted reproductive technology (ART) hormonal stimulation protocols have been developed to increase pregnancy and delivery rates in AMA women (Ubaldi et al.). One approach is mathematical—increase the likelihood of pregnancy by maximizing gonadotropin dose to produce a high yield of embryos. Other REI practices operate under the concept that high-dose FSH may adversely impact oocyte and/or embryo quality. Thus, they perform “mild” or “natural” IVF cycles employing low or no FSH administration. This results in lower embryo yield than the high dose protocols. There may be a “Goldilocks” dose for each patient that optimizes her yield of high-quality embryos.

### Embryo Selection

To date there are not established methods in humans for *preventing* oocyte and embryo aneuploidy. However, optimized stimulation protocols coupled with embryo karyotyping by preimplantation genetic testing (PGT-A) of biopsied embryos are employed to improve reproductive outcomes in AMA women. This is achieved by selecting euploid embryos for transfer while avoiding the transfer of aneuploid embryos (Poli et al.). The combination of IVF, embryo growth to the blastocyst stage, and biopsy of trophectoderm, are state-of-the-art practice by most IVF labs that perform PGT-A. Biopsying without damage to embryos requires specialized expertise not available to most IVF laboratories. Thus, evaluation of non-invasive procurement of chromosomal material from spent culture media to assess aneuploidy is under development. Procured samples are analyzed by a variety of means including PGT-A, and most recently MALDI-TOF mass spectrometry (Ubaldi et al.; Poli et al.) (10).

### Testing to Determine Embryo Karyotype

Comprehensive chromosomal screening (CCS) with Next Generation Sequencing (NGS) and Single Nucleotide Polymorphism (SNP) analyses of day 5–6 trophectoderm biopsies are state-of-the-art PGT-A methods employed to quantitate copy number for all 24 human chromosomes (Ubaldi et al.; Poli et al.). A study by Neal et al. compared a large group of PGT-A patients to a retrospectively defined patient cohort that had undergone blastocyst transfers without prior PGT-A (mean patient age 36.0 ± 4.3) (11). PGT-A did not improve cumulative pregnancy rates, but it did improve pregnancy rates per transfer (pregnancy rates per retrieval were not reported), and it reduced time to pregnancy and rates of miscarriage. Several randomized clinical trials (RCTs) have tested the efficacy of PGT-A for improving pregnancy outcomes. Among other studies, some cited improvements, and others cited no improvements or inconclusive results for diverse clinical endpoints (Ubaldi et al.) (12–14). An RCT from 2020 showed that IVF patients undergoing PGT-A had fewer cycles culminating in embryo transfers than those who had IVF alone (15). Despite this drawback, AMA patients (but not young patients) given PGT-A in the study had higher likelihood of clinical pregnancy and livebirth and lower probability of miscarriage than those who did not. Patients in a recent RCT for AMA women with severe DOR/POR showed no improvement of livebirth rates, and only nominal improvement in miscarriage rates (16). Additional RCTs are needed that include more patients and focus on defined patient cohorts—e.g. specific age groups; good/poor responders; recurrent miscarriage; repeated implantation failure. This will permit definitive conclusions regarding the efficacy of PGT-A in these diverse patient populations.

Development and testing of novel ancillary preimplantation embryo markers highly correlated with embryo quality and euploidy that are also underway, with the goal of selecting embryos that will increase pregnancy and livebirth rates. These include quantitative measurements of epigenetic landscape and transcriptomes (Poli et al.). Continuing advances are also being made in the realm of preimplantation genetic testing and selection of wild type embryos conceived by carriers of monogenic (PGT-M) and polygenic (PGT-P) mutations (Poli et al.; Treff et al.). PGT-M and PGT-P selection of wild-type embryos provides significant reduction in risks of disease inheritance. Coupled together, PGT-A, PGT-M, PGT-P, epigenetic, and transcriptosomal approaches hold promise to markedly improve the rates of healthy livebirths for patients at high risk of adverse outcomes due to maternal age and genetic disease mutations.

### Donor and Cryopreserved Eggs and Embryos

Health pregnancies for women of advanced maternal ages with severe DOR, POR and/or high aneuploidy rates in prior cycles are much more achievable using donated oocytes or emblryos from third parties (Poli et al.). Pregnancy and live birth rates are quite high, although gametes are from other people. Women who anticipate a need to delay pregnancy and wish to use their own eggs to conceive biological offspring can freeze their eggs or embryos for future pregnancy attempts, with increasingly promising success rates when oocytes are utilized within several years after oocyte retrieval.

### Experimental Therapies

Ovarian tissue banking is a developing technology for oocyte preservation until a future pregnancy is attempted (Ubaldi et al.). Nuclear transfer of spindle-chromosomal complexes, pronuclear or mitochondrial transfer, and chromosomal therapy are experimental approaches for improving reproductive parameters in infertile AMA patients. Their efficacies are not yet known. Generation of biologically “young” oocytes from putative oogonial stem cells (OSCs) has been under investigation for some time, but the existence of human OSCs has been called into question by investigators unable to reproduce OSC isolation protocols.

A review of detrimental effects of ROS in adversely impacting oocyte and embryo quality is provided by Máté et al. in this article collection. ROS has significant adverse impacts on spindle assembly and integrity. Various antioxidant treatments show effectiveness in animal studies to protect oocytes and embryos from spindle aberrations and aneuploidy, including resveratrol, N-acetyl cysteine, vitamins C and E, and other molecules. Melatonin is a potent antioxidant molecule that decreases mouse oocyte spindle aberrations, increases mitochondrial DNA copy number, and increases blastocyst quality. In human trials melatonin improved ovarian response and embryo quality compared to controls (17).

Coenzyme Q_10_ (CoQ_10_) has potent antioxidant activity. Also, it promotes mitochondrial ATP production in its capacity as a proton transport protein. CoQ_10_treatment restores mitochondrial function, cumulus cell function, and oocyte spindle integrity and fertility in reproductively aged female mice (18, 19). Infertile AMA IVF patients given CoQ_10_ trended toward increased embryo quality and decreased aneuploidy compared to the placebo group, although in this underpowered study the differences were not statistically significant (20). In patients <35 with DOR, women in the CoQ_10_ group had a better ovarian response and embryo quality than those in the placebo group, with statistically insignificant trends toward increased live birth and cumulative live birth rates (21). Similar treatments employing antioxidants and molecules that boost mitochondrial function are envisioned to treat endometriosis (Máté et al.).

Several approaches have been attempted and are under development to improve AMA fertility by correcting hormonal deficits. Mixed results have been obtained using therapies that employ growth hormone, dihydroepiandrosterone (DHEA), or testosterone (22, 23). As described above, activin receptor pathway blockade in midlife mice by administration of the activin decoy receptor ActRIIB:Fc lowers FSH to young levels, increases egg yield, prevents oocyte chromosome and spindle misalignments, and increases litter sizes (1). Manipulation of maternal hormone levels and pathways mediated by activin receptors provide promising avenues for developing new therapies to prevent oocyte and fetal aneuploidy and improve fertility in AMA women.

## Author Contributions

LB and NT wrote the manuscript. All authors contributed to the article and approved the submitted version.

## Conflict of Interest

LB is Founder and Chief Scientific Officer of Pregmama, LLC. NT is Founder of Genomic Prediction Inc. and Genomic Prediction Clinical Laboratory.
